# Charge Density-Based Pyroelectric Vacuum Sensor

**DOI:** 10.34133/research.0028

**Published:** 2023-01-16

**Authors:** Lan Xu, Geng Huangfu, Yiping Guo, Ya Yang

**Affiliations:** ^1^ CAS Center for Excellence in Nanoscience, Beijing Key Laboratory of Micro-nano Energy and Sensor, Beijing Institute of Nanoenergy and Nanosystems, Chinese Academy of Science, Beijing 101400, P.R. China.; ^2^ State Key Laboratory of Metal Matrix Composites, School of Materials Science and Engineering, Shanghai Jiao Tong University, Shanghai 200240, P.R. China.; ^3^ School of Nanoscience and Technology, University of Chinese Academy of Sciences, Beijing, 100049, P.R. China.; ^4^ School of Chemistry and Chemical Engineering, Center on Nanoenergy Research, Guangxi University, Nanning, Guangxi, 530004, P.R. China.

## Abstract

A traditional thermal conductivity vacuum gauge mainly detects low pressure (the degree of vacuum) by measuring the temperature change of a filament heated by the electric current. We propose a novel pyroelectric vacuum sensor that utilizes the effect of ambient thermal conductivity on the pyroelectric effect to detect vacuum through the charge density of ferroelectric materials under radiation. The functional relationship between the charge density and low pressure is derived, which is validated in a suspended (Pb,La)(Zr,Ti,Ni)O_3_ (PLZTN) ferroelectric ceramic-based device. The charge density of the indium tin oxide/PLZTN/Ag device under 405 nm of 60.5 mW cm^−2^ radiation at low pressure reaches 4.48 μC cm^−2^, which is increased by about 3.0 times compared with that at atmospheric pressure. The vacuum can improve the charge density without increasing the radiation energy, confirming the important role of ambient thermal conductivity on the pyroelectric effect. This research provides a demonstration for ambient thermal conductivity effectively tuning pyroelectric performance, a theoretical basis for pyroelectric vacuum sensors, and a feasible route for further optimizing the performance of pyroelectric photoelectric devices.

## Introduction

**Fig. 1.**
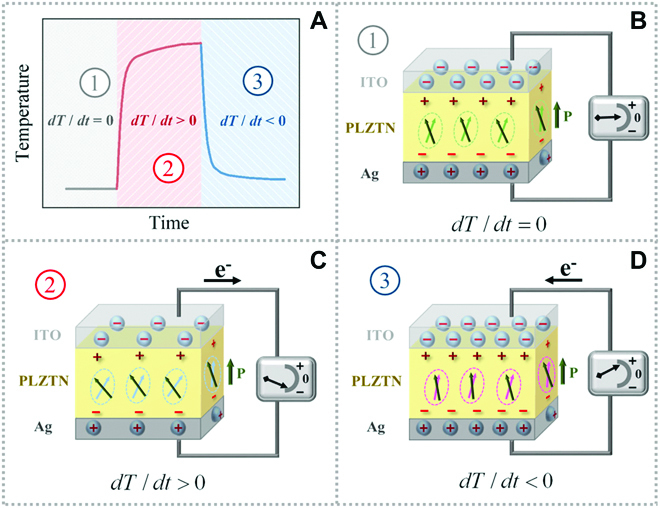
The photoinduced pyroelectric effect. (A to D) Schematic diagram of the pyroelectric effect and temperature changes in ferroelectric materials before (1), during (2), and after (3) sample irradiation. The physical mechanism: Bound charges in a polarized ferroelectric material appear near the sample surface, which is close to the 2 ends of the polarization vector. In the state of thermal equilibrium, these bound charges are shielded by the free charge with equal quantity but opposite sign, and no current is generated (B). When the light irradiation changes the sample temperature, the rising temperature will increase the oscillation of electric dipole and result in a decrease in polarization, thereby changing the ability to attract free charges. The reduction of the bound charge on the sample surface causes the redistribution of free charge and then generates an electric signal (C). After removing the stable light, the declined temperature increases the polarization of ferroelectric sample, and the increased free electrons form a pyroelectric electric signal (D).

In recent years, photodetectors and self-powered devices focusing on ferroelectric materials have attracted scientific interest, such as a pyroelectric nanogenerator using a typical ferroelectric material-lead zirconate titanate film, self-powered and ultraviolet detection with BaTiO_3_ ceramics as the core component, and a wavelength-selective pyroelectric infrared detector of LiTaO_3_ single crystal [1–3]. Photodetection on the basis of the pyroelectric effect has the advantages of ultrawide spectral response and the nonuse of cooling facilities [4]. Ferroelectric materials as an important subclass of pyroelectrics exhibit the reversible spontaneous polarization under an applied electric field. The physical mechanism of the photoinduced pyroelectric effect in ferroelectrics that converts light signals into electric signals is shown in Fig. 1. When a ferroelectric sample is suddenly exposed to or removed from the light illumination, the polarization changes with the temperature fluctuations, resulting in a redistribution of free charges, followed by a photoelectric response [5]. In this process, photoinduced temperature changes and charge density play important roles.

In pursuit of high performance, the design and modification of the material composition is a common and useful approach to optimize the pyroelectric properties. For instance, in the hybrid BaTiO_3_-Ag (silver) nanosystem, the photodeposition of Ag nanoparticles largely increased the surface charge density and achieved a 46% pyroelectric coefficient enhancement [6]. The introduction of ZnO into Bi_0.5_Na_0.5_TiO_3_-BaZr_0.2_Ti_0.8_O_3_ (BNT-BZT) ceramics improved its pyroelectric properties, thermal conductivity, and energy harvesting performance [7]. In addition, a ferroelectric poly(vinylidene difluoride)-based pyroelectric device chose graphene ink with high thermal radiation absorbance as one of the electrodes, which improved the pyroelectric current and the harvested energy [8]. However, the limitation of these research schemes is also obvious; that is, each material requires a targeted design, which apparently lacks universality. In the analysis of pyroelectric detectors, both pyroelectric properties and thermal conductivity should be considered. In the photoinduced pyroelectric effect, photoinduced temperature change is related not only to the radiation intensity but also to the heat dissipation of the sample. The medium surrounding the sample has a great influence on the thermal diffusion. Media with different thermal conductance will cause different photoinduced temperature change, thus affecting the electrical signal output. It is reported that a method of suspending the sample in a BaTiO_3_-based device achieves a high charge density of 1.7872 μC cm^−2^, which is almost 11.4 times compared with that of the device on substrate [9]. Their work corroborates important influence of thermal conductance of media on the electrical signal output in pyroelectric detectors, but the physical mechanism of the charge density enhancement still needs to be explored in depth. According to the reported thermal conductivity of various materials, it can be concluded that gases generally have low thermal conductivity [10–13]. Among them, air as a heat transfer medium has the advantage of being ubiquitous and readily available and it can be realized in practice by suspending the sample. Therefore, we set the medium around the sample to be gas, and then control ambient thermal conductivity around the sample through tuning the degree of vacuum, thereby modulating the pyroelectric output in real time. Conversely, if there is a functional relationship between the degree of vacuum and the pyroelectric output, it can be used to monitor the degree of vacuum.

**Fig. 2.**
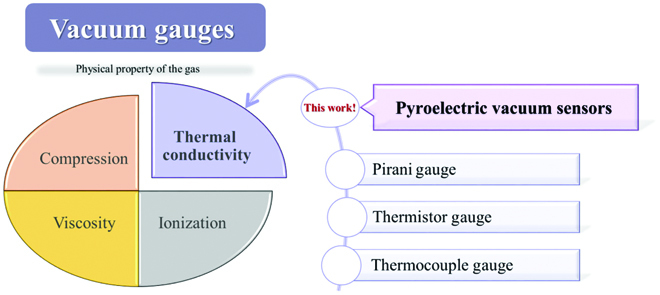
The classification of vacuum gauges according to the physical properties of the gas and the types of thermal conductivity vacuum gauges including the pyroelectric vacuum sensor (this work).

The degree of vacuum is mainly assessed by measuring the absolute pressure of a system, because the degree of vacuum increases as the pressure of the gas in a system decreases. The general definition of the term "vacuum" designates a given space filled with gas where pressure is less than atmospheric pressure (also called low pressure), and any device that monitors pressure in a vacuum system is defined as a vacuum gauge. Since no single vacuum gauge can sense the entire vacuum range, measurements can be made using a series of vacuum gauges, each of which responds within a specific pressure range. The types of vacuum gauges can be classified according to the physical properties of the gas, such as the pressure applied by the gas, the viscosity of the gas, momentum transfer rate, thermal conductivity, and ionization (Fig. 2) [14]. Traditional thermal conductivity vacuum gauges work by sensing the temperature change of the filaments associated with the heat loss caused by the conduction through the surrounding gas. A typical Pirani gauge and thermistor gauge measure the change in filament temperature with pressure through the change of the resistance, and a thermocouple gauge measures the temperature of filament by means of a thermocouple [14]. The thermal conductivity vacuum gauges have experienced an improvement from having large volumes and difficulty in mass production to miniaturization of gauges by introducing MEMS (Micro-Electro-Mechanical-Systems) technology [15–17]. However, the MEMS thermal conductivity gauges have complex structures, and the fabrication process of some devices may be incompatible with the CMOS (the complementary metal oxide semiconductor) process, which increases the cost of gauges and relatively reduces their performance [17]. Therefore, it is necessary to develop new types of vacuum sensors with a simple fabrication process. The pyroelectric vacuum sensor designed in this work mainly utilizes the relationship between the electric signal generated by the pyroelectric effect and the low pressure to detect the degree of vacuum, which is obviously different from the traditional thermal conductivity vacuum gauges. In addition, the fabrication of the device is very simple; one just needs to coat the pyroelectric element with electrodes. This work introduces huge materials with the pyroelectric effect into the vision of vacuum sensors, which plays an important role in the development of vacuum sensors.

The ferroelectric (Pb,La)(Zr,Ti)O_3_ system with outstanding ferroelectric, piezoelectric, and electro-optic properties and many constituents has been investigated in the waste heat energy harvesting originated from the pyroelectric effect [18–21]. As reported, the (Pb_0.955_La_0.03_)(Zr_0.86_Ti_0.14_)O_3_ ceramic with a high depolarization temperature of 179 °C displays a high pyroelectric coefficient of 520 μC m^−2^ K^−1^ and the figure of merit (FOM) for pyroelectric energy conversion can reach 60 J m^−3^ K^−2^ [22]. These pyroelectric properties are superior to modified lead zirconate titanate (PZT) piezoelectric ceramics, which has a pyroelectric coefficient of 380 μC m^−2^ K^−1^ and a FOM of 56 J m^−3^ K^−2^ [23,24]. In addition, the 0.75Pb(Mg_1/3_Nb_2/3_)O_3_-0.25PbTiO_3_ ceramic has a very high pyroelectric coefficient of 750 μC m^−2^ K^−1^ and a lower FOM of 30 J m^−3^ K^−2^ [25]. In this work, transparent Ni^2+^-doped (Pb,La)(Zr,Ti)O_3_ (abbreviated as PLZTN hereafter) ceramic shows an ultrahigh pyroelectric coefficient of 1,122 μC m^−2^ K^−1^ and a good FOM of 58 J m^−3^ K^−2^. Thus, PLZTN perovskite ferroelectric ceramics can be a good research platform to study the charge density of a pyroelectric device under radiation as a function of the degree of vacuum. The indium tin oxide (ITO)/PLZTN/Ag device with a suspended pyroelectric element structure can make the air around the pyroelectric element as the heat transfer medium, and ambient thermal conductivity can be adjusted by the degree of vacuum. The quantitative analysis reveals the important role of ambient thermal conductivity in the photoinduced pyroelectric effect. Based on our study, especially the derived functional relationship between the charge density and the degree of vacuum, we propose a new type of thermal conductivity vacuum gauge, namely, the pyroelectric vacuum sensor.

## Results

### The structure characterizations and electric properties of PLZTN and its device construction

**Fig. 3.**
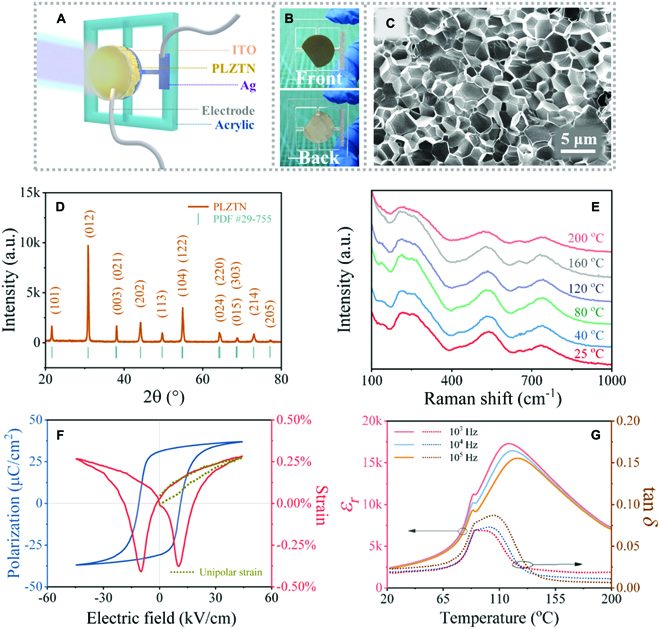
The structure characterization and electrical properties of the PLZTN ceramic and its pyroelectric detector. The schematic diagram of the ITO/PLZTN/Ag pyroelectric detector (A) and photographs for the front and back of the device (B). The structure and performance characterization of the PLZTN ceramic: (C) SEM micrograph of fracture surface, (D) XRD pattern indexed by PDF card #29-755, (E) the Raman spectra in different temperature, (F) the polarization–electric field (*P*–*E*) hysteresis loop and bipolar/unipolar electric-field-induced strain at room temperature with 10 Hz, and (G) the temperature dependence of dielectric constant *ɛ_r_* and dielectric loss tan *δ* at different frequencies.

Figure 3A displays the schematic diagram of the ITO/PLZTN/Ag pyroelectric device. The core sensing material, PLZTN ceramic, covered with the top electrode of transparent ITO and a bottom Ag electrode, forms an almost freestanding state as shown in Fig. 3B. The scanning electron microscope (SEM) micrograph of the fracture surface in Fig. 3C demonstrates that the ceramic has uniform crystal grains and a dense structure. Figure 3D shows the x-ray diffraction pattern of the PLZTN ceramic, revealing a rhombohedral *R*3*m* phase of lattice parameters of *a* = *b* = *c* = 4.09 Å and *α* = *β* = *γ* = 89.91°, which belongs to an ABO_3_-type perovskite structure. For further structure information, Fig. 3E shows the Raman spectra of the PLZTN ceramic at different temperatures in the range between 100 and 1,000 cm^−1^. These Raman peaks correspond to BO_6_ stretching/bending vibration modes and the A–O stretching mode [26,27]. It can be clearly seen that as the temperature increases, the Raman vibration modes gradually widen and weaken. It originates from the temperature-driven ferroelectric–paraelectric phase transition.

**Fig. 4.**
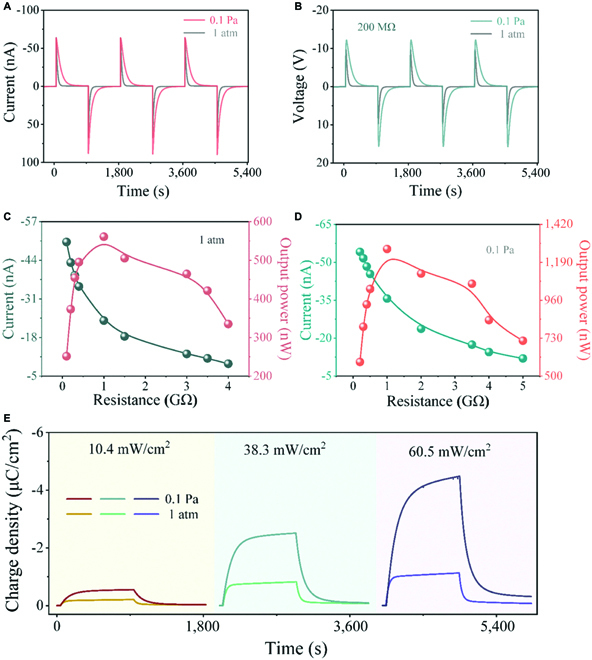
Pyroelectric output of the ITO/PLZTN/Ag device under 405-nm irradiation. (A) The output current under 405 nm of 38.3 mW cm^−2^ illumination at 1 atm and 0.1 Pa for the ITO/PLZTN/Ag device and (B) the voltage signals by parallel loading 200 MΩ. The loading resistance dependence of output current and output power at 1 atm (C) and 0.1 Pa (D) under 38.3 mW cm^−2^ illumination. (E) The charge density of the device at 1 atm and 0.1 Pa under 10.4, 38.3, and 60.5 mW cm^−2^ illumination.

Figure 3F displays a well-developed saturated ferroelectric hysteresis loop of the PLZTN ceramic and strain-electric field loops under strain drive (bipolar and unipolar). The coercive field (*E_c_*) is 11 kV cm^−1^ and the remnant polarization (*P_r_*) is 31 μC cm^−2^. The positive strain value can reach about 0.28%. The typical butterfly-like curve reflects the rotation of the ferroelectric domain driven by the bipolar field and also represents a good converse piezoelectric performance. The calculated piezoelectric constant from the unipolar strain curve is about 615 pm V^−1^. Figure 3G shows the temperature-dependent dielectric properties in the PLZTN ceramic. It can be clearly seen that the highest dielectric peak corresponding to Curie temperature indicating ferroelectric–paraelectric phase transition shifted from 117 °C at 10^3^ Hz to 125 °C at 10^5^ Hz, and this dielectric frequency dispersion represents a typical relaxor behavior. In addition, the transition from a nonergodic relaxor to an ergodic relaxor state occurs at the second dielectric peak, whose temperature (89 °C) is the depolarization temperature (*T*_d_) [28]. Below *T*_d_, the PLZTN material is in a nonergodic relaxor state, in which the electric field can induce an irreversible long-range ferroelectric order, so that high remnant polarization (Fig. 3F) and no obvious dielectric frequency dispersion (Fig. 3G) can be observed. When PLZTN is in an ergodic relaxor state above *T*_d_, in which the external field can induce a reversible ferroelectric phase, low remnant polarization in *P*–*E* curves and a sprout shape instead of a butterfly shape in *S*–*E* curves are displayed (Fig. S1), indicating the disappearance of long-range ordered ferroelectric domains in the relaxor state. The pyroelectric effect describes the change in spontaneous polarization induced by the relatively small temperature change, and for most ferroelectrics, the precondition is the polarized ferroelectric materials. Therefore, in principle, *T*_d_ should be the inflection point where the pyroelectric effect of relaxor ferroelectrics gradually disappears. The FOM for pyroelectric energy conversion is defined as *p_i_*^2^/*ε*_0_*ε_r_* (where *p_i_* is the pyroelectric coefficient, *ε*_0_ is the permittivity of free space, and *ε_r_* is the relative permittivity) [25]. That is to say, a high pyroelectric coefficient and a low dielectric constant are required for excellent pyroelectric energy harvesting, which are difficult to achieve simultaneously in general ferroelectric materials. The pyroelectric coefficient of the PZLTN ceramic in the suspended state calculated from Eq. 5 and Fig. 4E is 1,122 μC m^−2^ K^−1^ at 1 atm and the FOM is 58 J m^−3^ K^−2^ (*ε_r_* = 2,449 at 1 kHz), showing an excellent pyroelectric performance. In a word, the PLZTN ceramic has good ferroelectric, piezoelectric, and dielectric properties, making it a candidate for applications in pyroelectric photoelectric detectors, energy harvesting, and so on [29,30].

### The photoinduced pyroelectric performance at atmospheric and low pressure

We employed the substrate-free structure of Fig. 3A to measure the short-circuit currents and open-current voltages by 405-nm LED (light-emitting diode) excitation at a low pressure of 0.1 Pa and 1 atm, as shown in Fig. 4A and B and Fig. S2. It can be seen that under light intensities of 10.4, 38.3, and 60.5 mW cm^−2^, the output current at 0.1 Pa is higher than at 1 atm, but not much. For instance, the average of the current peaks under 38.3 mW cm^−2^ illumination is 63.8 nA at 0.1 Pa, while it is 63.7 nA at 1 atm (Fig. 4A). The pyroelectric current mainly depends on the change rate of sample temperature, as shown in Eq. 4, which is very small under different degrees of vacuum in our experimental conditions; thus, the influence of ambient thermal conductivity on the pyroelectric current is limited, resulting in small changes in the output current at different pressures. As shown in Fig. 4B, the average of the voltage peak is 12.2 V at 0.1 Pa, and the peak is 9.6 V at 1 atm. The influence of air pressure on the output voltage is greater than that of current, but the increase in voltage is obviously suppressed due to the load resistance. Furthermore, the output power of the ITO/PLZTN/Ag device loaded with a series of resistance at 1 atm and 0.1 Pa under 3 light intensities of 405-nm illumination is shown in Fig. 4C and D and Figs. S3 to S5. It can be seen that under 38.3 mW cm^−2^ illumination, the maximum output power can reach 1,271 nW by a loading resistance of 1 GΩ at 0.1 Pa, which is increased by about 1.3 times compared with that at 1 atm (Fig. 4C and D). Similar phenomena were also observed under 10.4 and 60.5 mW cm^−2^ illumination (Figs. S3C and D and S5C and D). Then, we explored the charge density generated from the ITO/PLZTN/Ag device under the 405-nm illumination with different intensities (Fig. 4E). The charge density of the ITO/PLZTN/Ag device at 0.1 Pa can reach 0.55 μC cm^−2^ under 10.4 mW cm^−2^ illumination, 2.51 μC cm^−2^ under 38.3 mW cm^−2^ illumination, and 4.48 μC cm^−2^ under 60.5 mW cm^−2^ illumination. Charge density at 0.1 Pa is increased by about 1.6 times under 10.4 mW cm^−2^ illumination, 2.1 times under 38.3 mW cm^−2^ illumination, and 3.0 times under 60.5 mW cm^−2^ illumination, compared with that at 1 atm, respectively. Obviously, the charge density of the ITO/PLZTN/Ag device under a low pressure of 0.1 Pa is much higher than that under atmosphere pressure, because the increase in charge density is mainly related to the temperature change of the sample. These experimental results reveal that in the photoinduced pyroelectric output signals, the charge density is the most sensitive to low pressure, and in detail, the enhancement of vacuum (1 atm ➔ 0.1 Pa) results in a marked increase in charge density.

### Quantitative analysis of charge density under vacuum

**Fig. 5.**
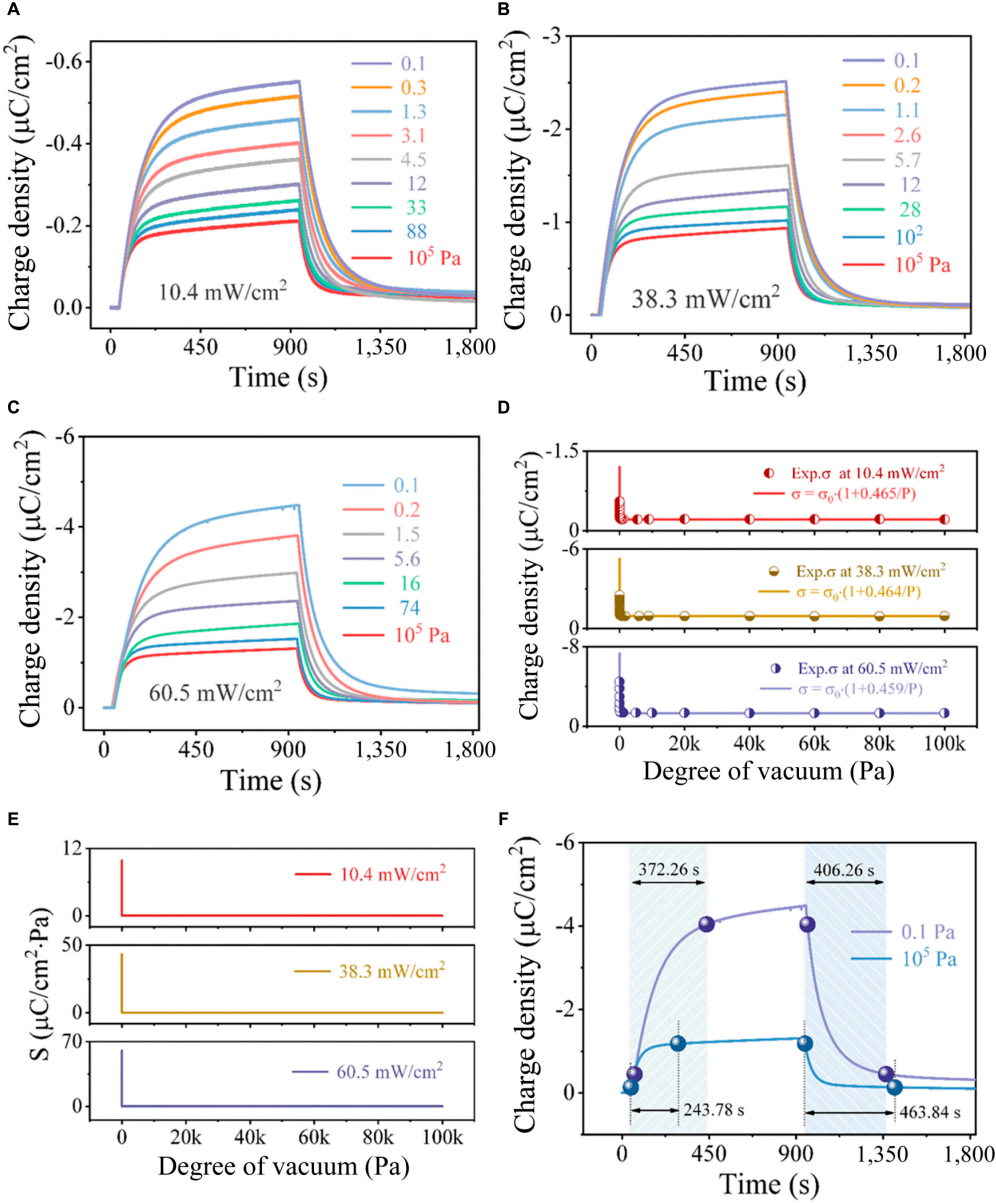
Experimental and theoretical values of the charge density of the ITO/PLZTN/Ag detector under different degrees of vacuum and the performance parameters. The charge density of the ITO/PLZTN/Ag device at different degrees of vacuum under a 405-nm irradiation of 10.4 mW cm^−2^ (A), 38.3 mW cm^−2^ (B), and 60.5 mW cm^−2^ (C). (D) The experimental data from 0.1 Pa to 10^5^ Pa and the curves from Eqs. 11 to 13 of the maximum charge density. (E) The degree of vacuum dependence of sensitivity from Eqs. 14 to 16 under 10.4 mW cm^−2^, 38.3 mW cm^−2^, and 60.5 mW cm^−2^ radiation. (F) The response speed of charge density under a 405-nm illumination of 60.5 mW cm^−2^ at 0.1 Pa and 10^5^ Pa.

A detailed study on the charge density under different degrees of vacuum was conducted. Figure 5A to C displays the charge densities of the ITO/PLZTN/Ag device irradiating by 405 nm with 3 light intensities at a low pressure ranging from 10^5^ to 0.1 Pa. Extracted from Fig. 5A to C and the corresponding charge in Fig. S6, the maximum charge density as a function of the degree of vacuum is shown in Fig. 5D. In the case of 405-nm illumination with a constant light intensity, the charge density is related to the degree of vacuum with the most sensitive range between 100 and 0.1 Pa, which conforms to thermal conductivity vacuum gauges [31]. In the photoinduced pyroelectric output, the determinant temperature variation of a pyroelectric element is closely linked to the thermal conductivity of the element to the surrounding environment. Hence, we adopted the suspended pyroelectric element to make the heat transfer medium around the element mainly air. The change in the charge density of the ITO/PLZTN/Ag device with the degree of vacuum is mainly realized by the variation of thermal conductivity of the heat transfer medium air. It is well known that the change in thermal conductivity of gas with pressure is the physical basis for thermal conductivity vacuum gauges.

**Fig. 6.**
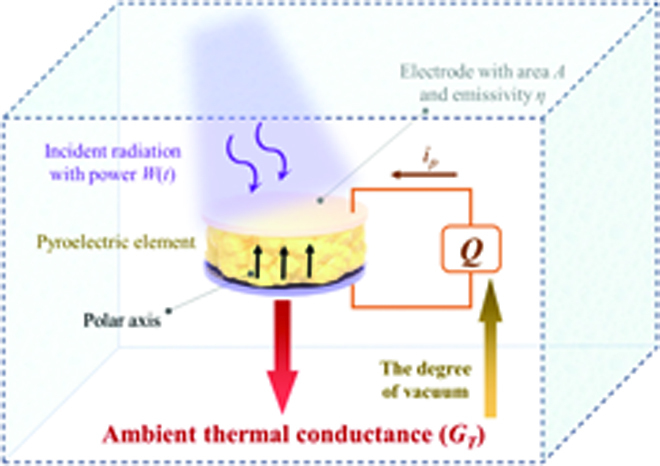
Schematic diagram of a photoinduced pyroelectric detector in a specific space.

Figure 6 shows the thermal conductivity and simplified electrical circuit sections of a photoinduced pyroelectric detector in a specific space. If radiation *W*(*t*) with periodic frequency *ω* is incident normal to the surface of the pyroelectric element, whose effective emissivity is *η*, and induces a temperature change Δ*T*, it is described as

$$\eta W\left(t\right)=H\frac{d\left(\Delta T\right)}{\mathrm{dt}}+G_{T}\Delta T,$$ (1)

where *H* and *G_T_* are the element thermal capacity and the thermal conductance to the surrounding environment (or called ambient thermal conductivity), respectively [29,32]. The solution of Eq. 1 is

$$\Delta T=\frac{\eta W\left(t\right)}{G_{T}+\mathrm{j\omega H}}\;.$$ (2)

The pyroelectric effect refers to the spontaneous polarization ***P***_***s***_ with the change of temperature (*T*), which is expressed by the pyroelectric coefficient ***p*** (a vector) as [29]

$$p=\frac{\Delta P_{s}}{\Delta T}\;.$$ (3)

When a thin plate of polarized ferroelectric covered with electrode area *A* on the upper and lower surfaces is selected as the pyroelectric element, the pyroelectric charge (*Q*) and the pyroelectric current *i_p_* generated in an external circuit has the form

$$i_{p}=\frac{\mathrm{dQ}}{\mathrm{dt}}={\mathrm{Ap}}_{i}\frac{\mathrm{dT}}{\mathrm{dt}}\;,$$ (4)

where *p_i_* is a component of vector ***p*** normal to the sample surface (Fig. 1) [29]. Integrating Eq. 4 with respect to *t* (time) generates charge density

$$\sigma =\frac{\Delta Q}{A}=p_{i}\Delta T\;.$$ (5)

The substitution of Eq. 2 into Eq. 5 gives

$$\sigma =\frac{\eta p_{i}W\left(t\right)}{G_{T}+\mathrm{j\omega H}}\;.$$ (6)

Since *H* and *p_i_* are the physical properties of the pyroelectric, the charge density generated in the photoinduced pyroelectric effect is mainly affected by *G_T_* in the case of the given element, ensuring the same radiation. Thus, in theory, the charge density can be enhanced by lowering ambient thermal conductivity.

According to reports [33,34], the thermal conductivity of air in closed space can be approximately expressed as

$$\kappa_{\mathrm{air}}=\kappa_{o}\times \frac{1}{1+{\frac{7.6\times 10}{P\times \frac{D}{T_{\mathrm{avg}}}}}^{-5}}\left({\mathrm{Wm}}^{-1},K^{-1}\right),$$ (7)

where *κ_o_* is the thermal conductivity of air at atmosphere pressure and room temperature, *T_avg_* represents the average temperature of enclosed space, *D* is the distance between the element and heat sink, and *P* denotes the pressure of air. If the suspended element within a closed cavity is irradiated with a constant light power *W* for a period time at room temperature (*ω* = 0), substituting the thermal conductivity of air *κ_air_* given by Eq. 7 when the thermal conductance *G_T_* only considers *κ_air_* (*G_T_* = *κ_air_*) into Eq. 6 generates

$$\sigma =\frac{\eta p_{i}W}{\kappa_{o}}\cdot \left(1+{\frac{7.6\times 10}{P\times \frac{D}{T_{\mathrm{avg}}}}}^{-5}\right)\;.$$ (8)

At room temperature and atmosphere pressure, the corresponding charge density is

$$\sigma_{0}=\frac{\eta p_{i}W}{\kappa_{o}}\;.$$ (9)

Substituting Eq. 9 into Eq. 8, the charge density is now expressed as

$$\sigma =\sigma_{0}\cdot \left(1+{\frac{7.6\times 10}{P\times \frac{D}{T_{\mathrm{avg}}}}}^{-5}\right)\;.$$ (10)

Therefore, through the abbreviated Eq. 10, the charge density changes with air pressure according to the functional relationship of $\sigma =\sigma_{0}\cdot \left(1+\frac{N}{P}\right)$ (*N* is a constant) at a specified temperature. It is the theory basis of pyroelectric vacuum sensors and vacuum enhancement of charge density in the photoinduced pyroelectric effect. Additionally, the relationship between output voltage, current, and power with air pressure is discussed in Supplementary Note 1.

Incorporating the detector parameters (in Materials and Methods) into Eq. 10, the charge density of the ITO/PLZTN/Ag device as a function of the degree of vacuum by irradiating at 405 nm with light intensities of 10.4, 38.3, and 60.5 mW cm^−2^ can be respectively expressed as

$$\sigma_{1}=\sigma_{0[10.4\;\mathrm{mW}/{\mathrm{cm}}^{2}]}\cdot (1+0.465/P),$$ (11)

$$\sigma_{2}=\sigma_{0[38.3\;\mathrm{mW}/{\mathrm{cm}}^{2}]}\cdot (1+0.464/P),$$ (12)

$$\sigma_{3}=\sigma_{0[60.5\;\mathrm{mW}/{\mathrm{cm}}^{2}]}\cdot (1+0.459/P).$$ (13)

The curves in Fig. 5D, obtained from Eqs. 11 to 13, and the experimental data of the maximum charge density (Fig. 5A to C and Fig. S6) are good consistent with each other in the range from 10^5^ to 0.1 Pa.

### The device performance

To evaluate the performance of the detector, sensitivity and response speed are important parameters. It is known that the sensitivity of a linear sensor is a constant, but for a nonlinear sensor like the device in this work, the sensitivity *S* is a variable in the form of *S* = *dy*/*dx*, and the sensitivity of the ITO/PLZTN/Ag device is calculated from the derivative of Eqs. 11 to 13 with respect to pressure *P*, expressed as

$$S_{1[10.4\;\mathrm{mW}/{\mathrm{cm}}^{2}]}={\left(\sigma_{1}\right)}^{\prime }=\frac{0.099}{P^{2}}(\mu C/{\mathrm{cm}}^{2}),$$ (14)

$$S_{2[38.3\;\mathrm{mW}/{\mathrm{cm}}^{2}]}={\left(\sigma_{2}\right)}^{\prime }=\frac{0.434}{P^{2}}(\mu C/{\mathrm{cm}}^{2}),$$ (15)

$$S_{3[60.5\;\mathrm{mW}/{\mathrm{cm}}^{2}]}={\left(\sigma_{3}\right)}^{\prime }=\frac{0.602}{P^{2}}(\mu C/{\mathrm{cm}}^{2}).$$ (16)

Figure 5E shows the sensitivity *S* as a function of pressure *P* according to Eqs. 14 to 16 at 3 irradiation intensities, indicating that the higher the degree of vacuum, the greater the sensitivity. In addition, the response speed of charge density under 405-nm illumination is shown in Fig. 5F and Fig. S7. Here, the response speed is expressed by the rise time and decay time, that is, the time required for the maximum charge density rising from 10% to 90% under radiation, and the time needed for the maximum charge density declining from 90% to 10% after the light is removed [35]. The charts in Fig. S7C to E show that the rise time is slightly affected by the degree of vacuum and is within a certain range of values in this experimental condition. As previously mentioned, the pyroelectric signal is produced under sudden irradiation. When constant irradiation continues, the temperature of the sample will eventually reach a new steady value, and the charge density in the circuit will tend to be stable in the circuit. According to Eqs. 5 and 2, the charge density of a sample is related to the temperature change induced by the radiation, so the corresponding response speed depends on the thermal relaxation time of the pyroelectric element [36], where *H*/*G*_T_ is a thermal time constant [29]. It is reported that a BaTiO_3_-based pyroelectric device has a faster response time on the substrate than those in suspended state, which is mainly due to the difference in the thermal conductance to the surrounding environment (*G*_T_) [9]. The response time of the Pb(Zr,Ti)O_3_ (PZT) film device is 10 times shorter than that of the PZT ceramic device, because the thermal capacity (*H*) of the PZT film device is less than that of the ceramic device [37]. In addition, different radiation sources also have an impact on the response speed. When the PLZTN device is exposed to a laser irradiation with a faster response time than LED, the response speed has been significantly improved, as shown in Fig. S8. It can also be seen that the response speed at 1 atm is obviously faster than that at 0.1 Pa, due to the influence of *G*_T_.

Furthermore, as shown in Fig. S1, PLZTN ceramics display good and stable ferroelectric and converse piezoelectric properties below a depolarization temperature of 89 °C, which is a good prerequisite for pyroelectric output. It can be seen that the charge density of the PLZTN device has good temperature stability from room temperature to about 50 °C (Fig. S9). From the perspective of expanding the range of temperature stability, we think that ferroelectric materials with higher Curie temperature (such as LiTaO_3_ with a *T*_C_ of 665 °C [29]) are good alternatives as components of a pyroelectric vacuum detector. Besides, as shown in Fig. S10, when the temperature is above the depolarization temperature of 89 °C, the pyroelectric effect of PLZTN disappears. Thus, the PLZTN pyroelectric vacuum sensor cannot work beyond depolarization temperature. In addition, Fig. S11 records the charge density of the PLZTN device at different times under periodic irradiation lasting 12 h, demonstrating good performance stability. As mentioned above, the charge density has a functional relationship with the degree of vacuum of air in the form of $\sigma =\sigma_{0}\cdot \left(1+\frac{N}{P}\right)$ from 10^5^ to 0.1 Pa in the photoinduced pyroelectric effect. Therefore, the charge density of the pyroelectric element under radiation can be used to detect the degree of vacuum, and its essence is to sense ambient thermal conductivity at different low pressures. In a pyroelectric vacuum sensor, the lighting equipment can be installed outside the vacuum system, which is easy to disassemble and carry, and the device can realize self-powered electrical signal output without requiring additional power during operation. In actual use, it is necessary to further optimize the pyroelectric device, correct the parameters of vacuum detection, and perform calibration.

## Discussion

We proposed a novel charge density–pyroelectric vacuum sensor following the formula $\sigma =\sigma_{0}\cdot \left(1+\frac{N}{P}\right)$, which is verified on the ITO/PLZTN/Ag device with suspended pyroelectric element configuration. It is indeed feasible to manipulate pyroelectric performance by controlling ambient thermal conductivity. The pyroelectric devices applied to photodetectors can greatly increase the charge density by improving the degree of vacuum around the pyroelectric element, which can be achieved by sealing and vacuuming the device. The charge density of the ITO/PLZTN/Ag device at low pressure can reach 4.48 μC cm^−2^ under 60.5 mW cm^−2^, which is increased by about 3.0 times of that at atmospheric pressure and room temperature. This method of greatly improving the performance of devices without changing the material itself is expected to be applied to most pyroelectric devices and has great advantages in performance optimization.

## Materials and Methods

(Pb_0.91_La_0.09_)(Zr_0.634_Ti_0.341_Ni_0.025_)O_3_ (abbreviated as PLZTN) ceramics were prepared by the solid-state reaction method and hot-pressing technique [38]. The sample was cut into small pieces that are 0.3 mm thick. The crystalline structure was measured by x-ray powder diffraction (D8 ADVANCE, Bruker) with Cu K_α_ radiation. The micrograph of fracture surface was performed using a field emission SEM (Nova NanoSEM 450). Raman scattering experiments at different temperatures were performed on a micro-Raman spectrometer (Lab-Ram HR Evolution, Horiba) equipped with a Linkam THMSE 600 heating stage, using a 514-nm laser as an exciting source. A ferroelectric tester (TF Analyzer 1000, aixACCT) was employed to test the polarization–electric field hysteresis loop and electric-field-induced strain. Dielectric properties were tested by using an impedance analyzer (Concept 40, Novocontrol). The transparent ITO and Ag electrodes were coated on the top and bottom surfaces of the ceramic plate using the magnetron sputtering method for photoelectric measurements. The electrode area of the sample is 3.24 cm^2^. The output current and voltage signals of the PLZTN device were determined using the 2611B system sourcemeter (Keithley). A Keithley 6514 system electrometer was used to measure the output charge. When measuring the photoelectric signals at different pressures, the ITO/PLZTN/Ag device was placed in a closed cavity, and the distance between the device and the cavity is 4.908 cm.

## Data Availability

All data that support the findings of this study are available from the corresponding author upon reasonable request.

## Supplementary Materials

Supplementary Materials Fig. S1. The *P*–*E* and *S*–*E* curves of PLZTN ceramics at different temperatures. Fig. S2. Pyroelectric output current and voltage of the ITO/PLZTN/Ag device under 405-nm irradiation. Fig. S3. Output currents by loading with series resistance and output power under a 405 nm-illumination of 10.4 mW cm^−2^. Fig. S4. Output currents by loading with different series resistance at 1 atm (A) and 0.1 Pa (B) under a 405-nm illumination of 38.3 mW cm^−2^. Fig. S5. Output currents by loading with series resistance and output power under a 405-nm illumination of 60.5 mW cm^−2^. Fig. S6. The detailed charge of the ITO/PLZTN/Ag device at different pressures under a 405-nm irradiation of 10.4 mW cm^−2^ (A), 38.3 mW cm^−2^ (B), and 60.5 mW cm^−2^ (C). Fig. S7. The response speed of charge density under a 405-nm illumination of 10.4 mW cm^−2^ (A, C), 38.3 mW cm^−2^ (B, D), and 60.5 mW cm^−2^ (E) at different degrees of vacuum. Fig. S8. The response speed of charge density under a 405-nm laser illumination of 60.5 mW cm^−1^ at 0.1 Pa and 1 atm. Fig. S9. Charge density of the PLZTN device at different temperatures at 1 atm and 0.1 Pa. Fig. S10. Charge density of the PLZTN device at 24 and 92 °C under 1 atm. Fig. S11. Charge density of the PLZTN device at 0, 3, 6, 9, and 12 h during continuous periodic irradiation for 12 h under 1 atm and 0.1 Pa, respectively. Supplementary Note 1. The relationship of output voltage, current, and power with air pressure (Supplementary Materials).
