# Supplementary material for: Charge Density-Based Pyroelectric Vacuum Sensor
Source: Research (Wash D C). 2023 Jan 16;6:0028. doi: 10.34133/research.0028 (PMC10076003; doi:10.34133/research.0028)
Supplement: Supplementary Materials — Fig. S1. The P–E and S–E curves of PLZTN ceramics at different temperatures. Fig. S2. Pyroelectric output current and voltage of the ITO/PLZTN/Ag device under 405-nm irradiation. Fig. S3. Output currents by loading with series resistance and output power under a 405 nm-illumination of 10.4 mW cm−2. Fig. S4. Output currents by loading with different series resistance at 1 atm (A) and 0.1 Pa (B) under a 405-nm illumination of 38.3 mW cm−2. Fig. S5. Output currents by loading with series resistance and output power under a 405-nm illumination of 60.5 mW cm−2. Fig. S6. The detailed charge of the ITO/PLZTN/Ag device at different pressures under a 405-nm irradiation of 10.4 mW cm−2 (A), 38.3 mW cm−2 (B), and 60.5 mW cm−2 (C). Fig. S7. The response speed of charge density under a 405-nm illumination of 10.4 mW cm−2 (A, C), 38.3 mW cm−2 (B, D), and 60.5 mW cm−2 (E) at different degrees of vacuum. Fig. S8. The response speed of charge density under a 405-nm laser illumination of 60.5 mW cm−1 at 0.1 Pa and 1 atm. Fig. S9. Charge density of the PLZTN device at different temperatures at 1 atm and 0.1 Pa. Fig. S10. Charge density of the PLZTN device at 24 and 92 °C under 1 atm. Fig. S11. Charge density of the PLZTN device at 0, 3, 6, 9, and 12 h during continuous periodic irradiation for 12 h under 1 atm and 0.1 Pa, respectively. Supplementary Note 1. The relationship of output voltage, current, and power with air pressure (Supplementary Materials). [file research.0028.f1.docx]

Supplementary Materials

**Title**

**Charge Density-Based Pyroelectric Vacuum Sensor**

**Authors**

Lan Xu^1^†, Geng Huangfu^2^†, Yiping Guo^2^* and Ya Yang^1,3,4*^

**Affiliations**

^1^CAS Center for Excellence in Nanoscience, Beijing Key Laboratory of Micro-nano Energy and Sensor, Beijing Institute of Nanoenergy and Nanosystems, Chinese Academy of Science, Beijing 101400, P.R. China.

^2^State Key Laboratory of Metal Matrix Composites, School of Materials Science and Engineering, Shanghai Jiao Tong University, Shanghai 200240, P. R. China

^3^School of Nanoscience and Technology, University of Chinese Academy of Sciences, Beijing, 100049, P.R. China.

^4^School of Chemistry and Chemical Engineering, Center on Nanoenergy Research, Guangxi University, Nanning, Guangxi, 530004, P.R. China

Correspondence should be addressed to Ya Yang: yayang@binn.cas.cn: Yiping Guo: ypguo@sjtu.edu.cn

† These authors contributed equally to this work.


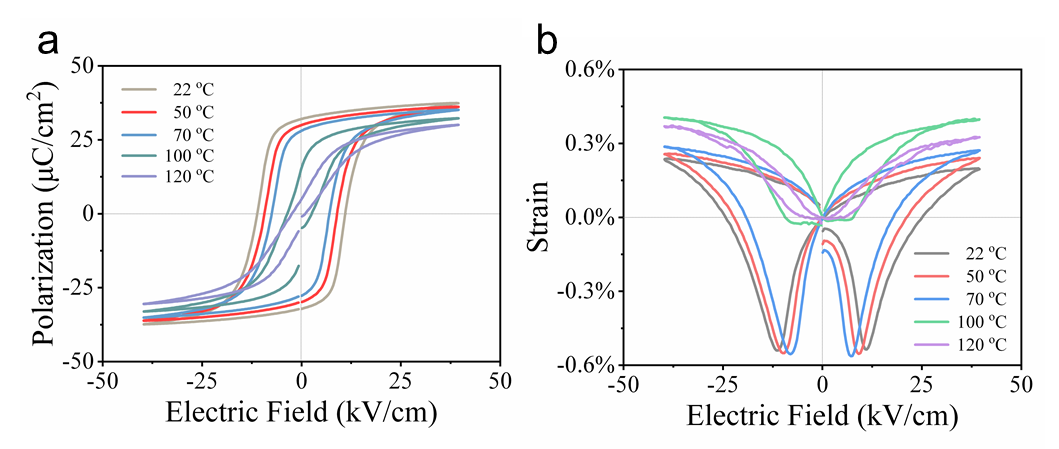


**Figure S1:** The *P*-*E* and *S*-*E* curves of PLZTN ceramics at different temperatures.


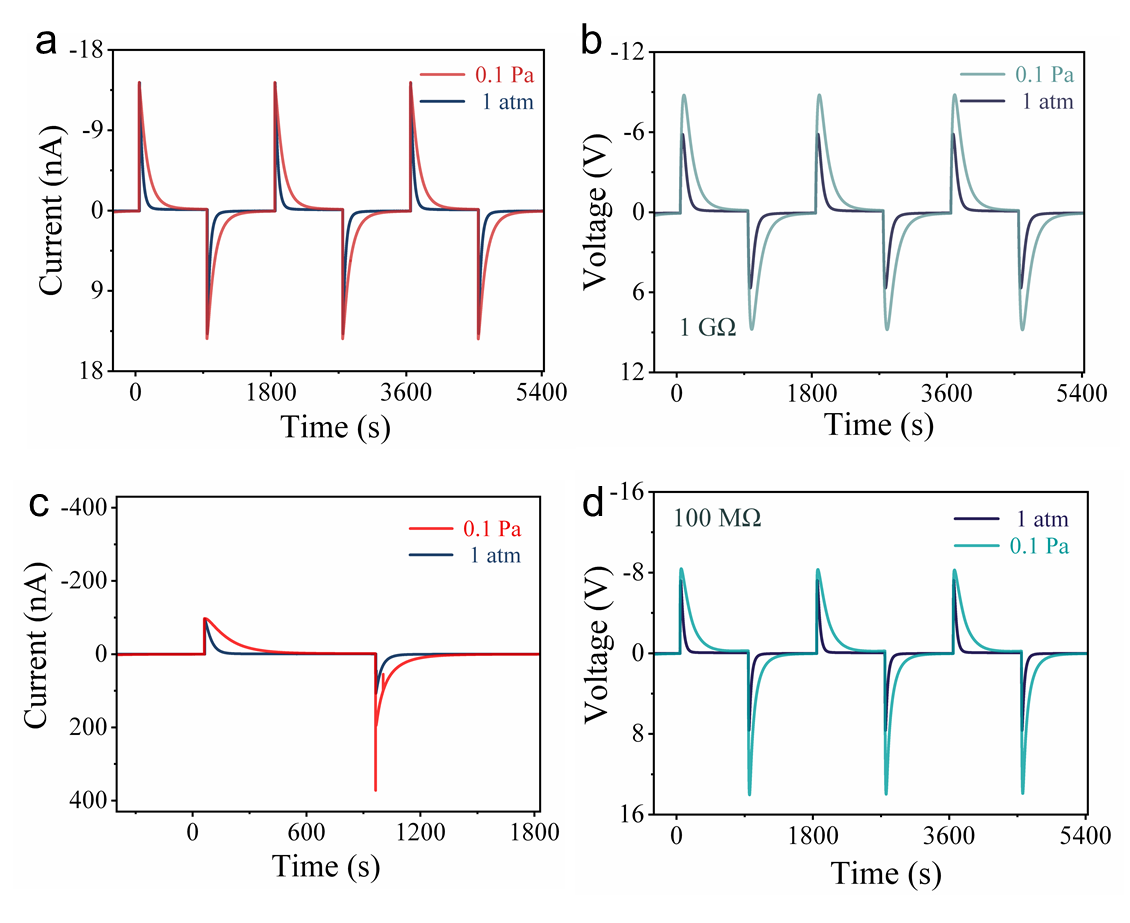


**Figure S2:** Pyroelectric output current and voltage of ITO/PLZTN/Ag device under 405 nm irradiation. (a) The output current under 405 nm of 10.4 mW cm^-2^ illumination at 1 atm and 0.1 Pa for ITO/PLZTN/Ag device and (b) the voltage signals by parallel loading 1 GΩ. (c) The output current under 60.5 mW cm^-2^ illumination at 1 atm and 0.1 Pa and (d) the voltage signals by parallel loading 100 MΩ.


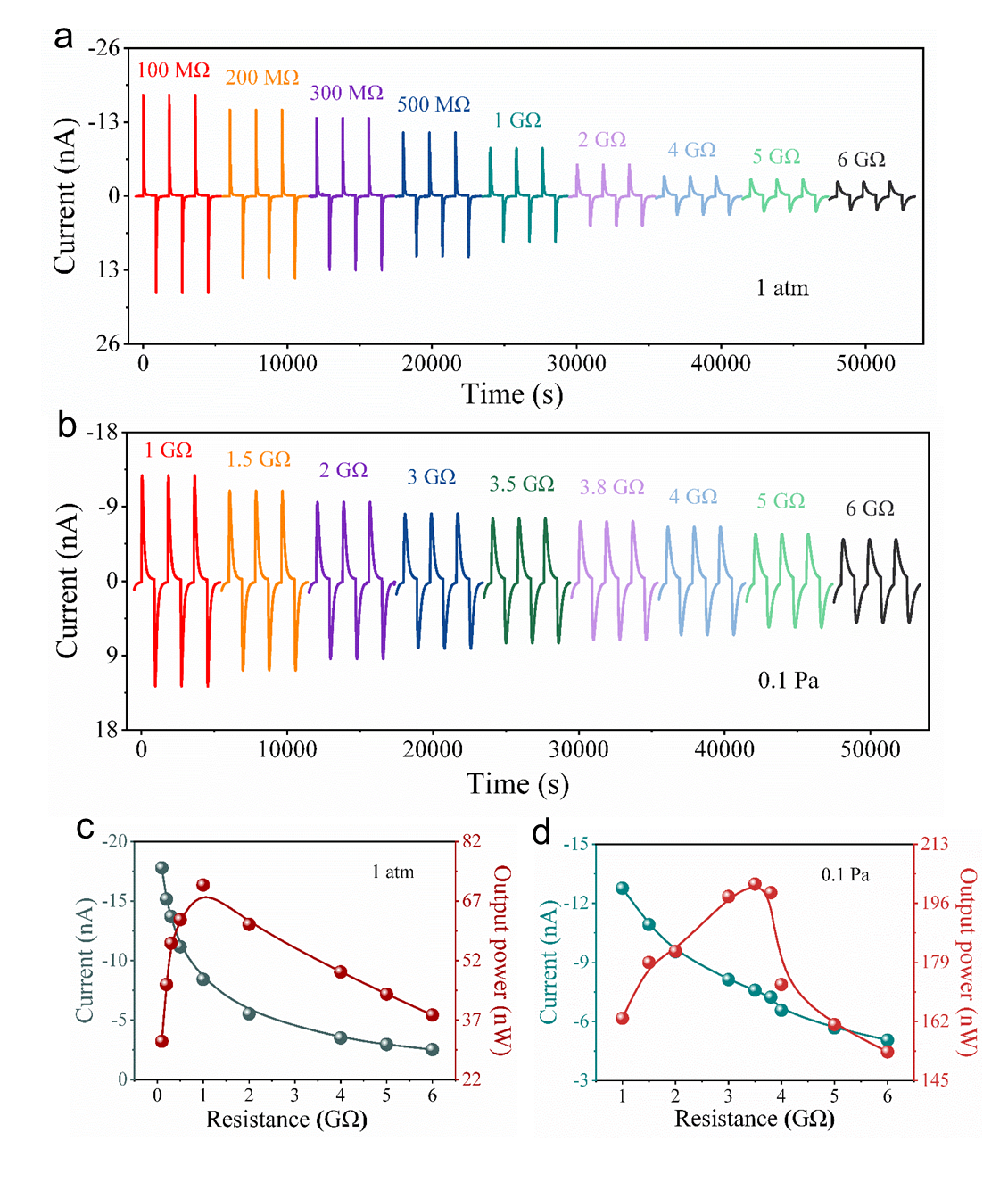


**Figure S3:** Output currents by loading with series resistance and output power under 405 nm illumination of 10.4 mW cm^-2^. Output currents by loading with different series resistance at 1 atm (a) and 0.1 Pa (b) under 405 nm illumination of 10.4 mW cm^-2^. The output current and output power as a function of loading resistance at 1 atm (c) and 0.1 Pa (d). The maximum output power at 0.1 Pa is increased by about 1.8 times compared with that at 1 atm. The area of this sample is 3.68 cm^2^.


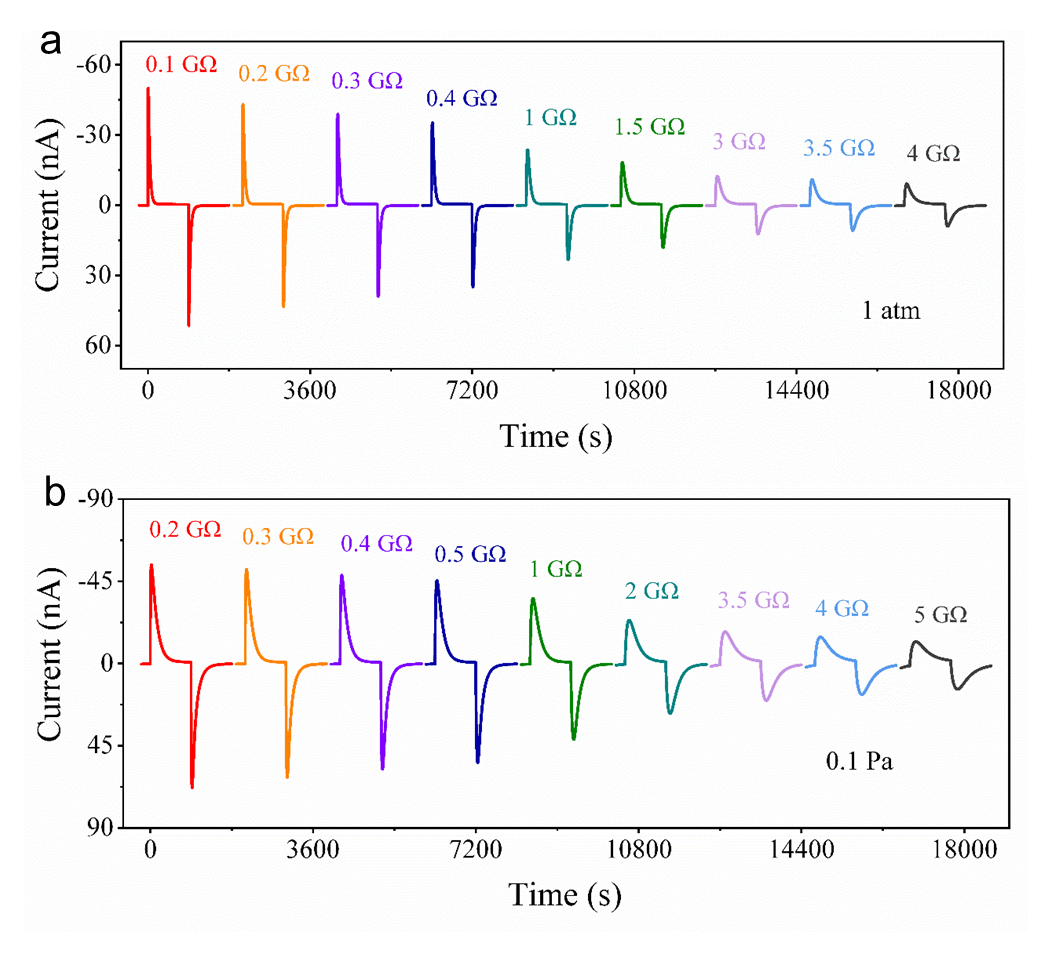


**Figure S4:** Output currents by loading with different series resistance at 1 atm (a) and 0.1 Pa (b) under 405 nm illumination of 38.3 mW cm^-2^.


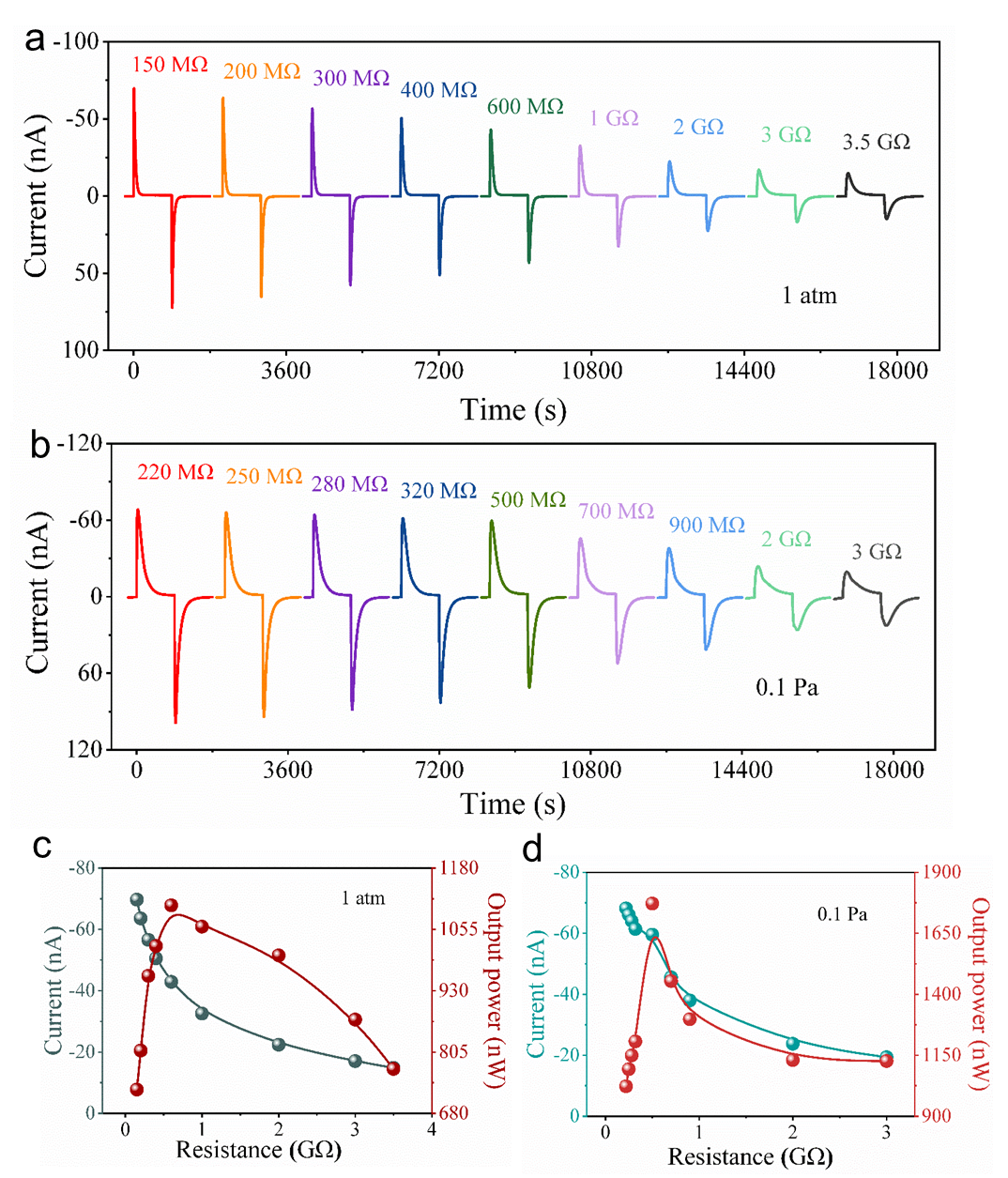


**Figure S5:** Output currents by loading with series resistance and output power under 405 nm illumination of 60.5 mWcm^-2^. Output currents by loading with different series resistance at 1 atm (a) and 0.1 Pa (b) under 405 nm illumination of 60.5 mW cm^-2^. The output current and output power as a function of loading resistance at 1 atm (c) and 0.1 Pa (d). The maximum output power at 0.1 Pa is increased by about 0.6 times compared with that at 1 atm.


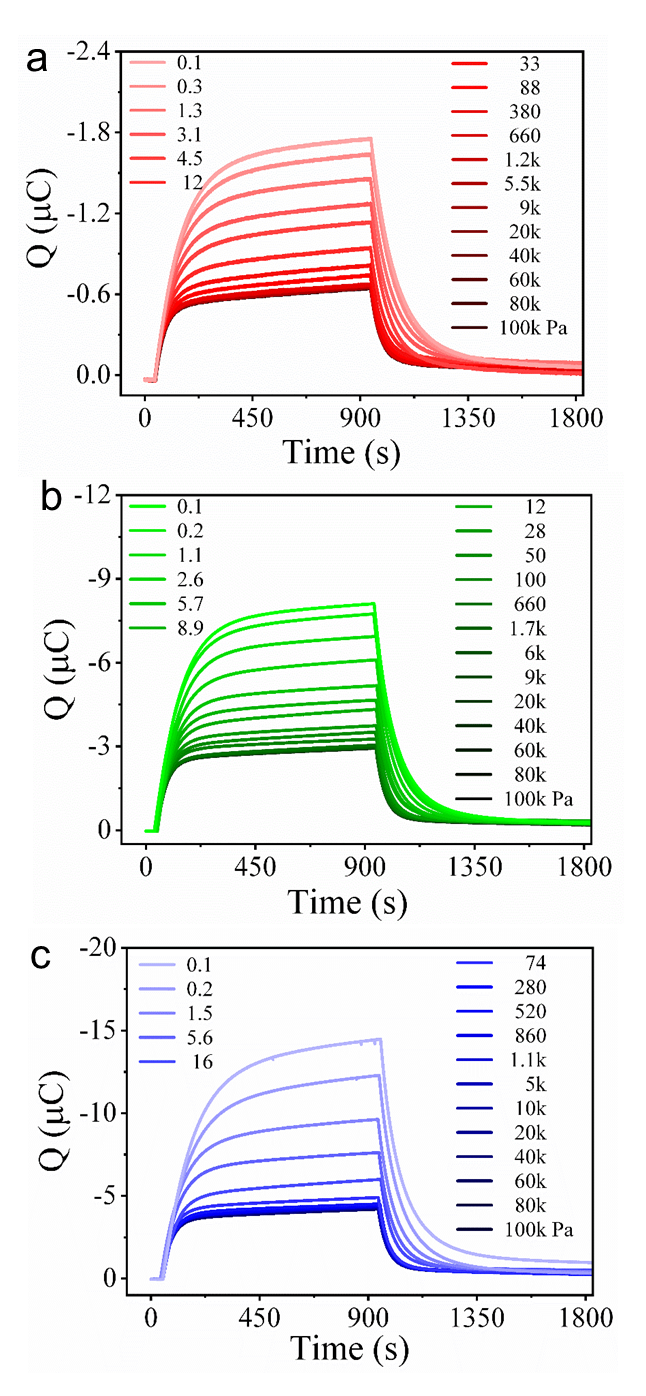


**Figure S6:** The detailed charge of ITO/PLZTN/Ag device at different pressure under 405 nm irradiation of 10.4 mWcm^-2^ (a), 38.3 mW cm^-2^ (b) and 60.5 mW cm^-2^ (c).


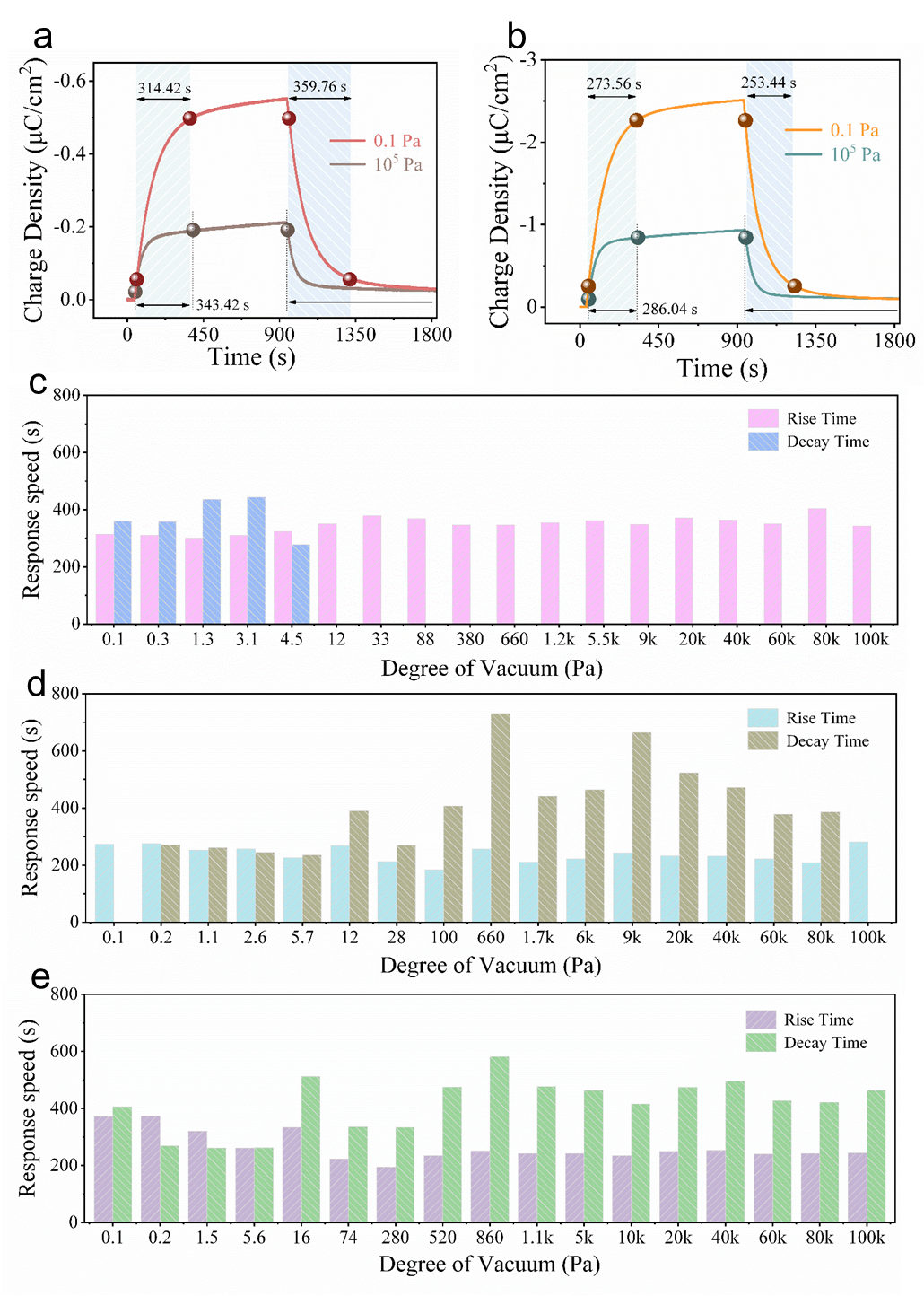


**Figure S7:** The response speed of charge density under 405 nm illumination of 10.4 mW cm^-2^ (a, c), 38.3 mW cm^-2^ (b, d) and 60.5 mW cm^-2^ (e) at different degree of vacuum.





**Figure S8:** The response speed of charge density under 405 nm laser illumination of 60.5 mW cm^-1^ at 0.1 Pa and 1 atm. The spot area of the laser is 0.049 cm^2^.





**Figure S9:** Charge density of PLZTN device under different temperature at 1 atm and 0.1 Pa. The area of this sample is 1.01 cm^2^.





**Figure S10:** Charge density of PLZTN device at 24 ^o^C and 92 ^o^C under 1 atm. The area of this sample is 0.77 cm^2^.





**Figure S11:** Charge density of PLZTN device at 0 h, 3 h, 6 h, 9 h and 12 h during continuous periodic irradiation for 12 hours under 1 atm and 0.1 Pa, respectively. The area of this sample is 1.01 cm^2^.

**Supplementary Note 1: The relationship of output voltage, current and power with air pressure.**

Based on the section 2.3, as a polarized ferroelectric material is a kind of dielectric, the open circuit voltage (*V*) developed in an external circuit should satisfy the formula:

$V=\frac{\Delta Q}{C}$, (S1)

where *C* is the equivalent capacitance, defined as $C=A\varepsilon/h$. The open circuit voltage, from Equation (S1) and (5), also has the form [1]:

$V=\frac{p_{i}}{\varepsilon}\text{ }h\text{ }\Delta T$. (S2)

The thickness of ferroelectric sample is *h*, and the permittivity is *ε*. Substituting Equation (5) and (10) into Equation (S1), the open circuit voltage can be expressed as:

$V=V_{0}\cdot\left(1+\frac{7.6\times10}{P\times\frac{D}{T_{avg}}}^{-5}\right)$, (S3)

where *V*_0_ is the open circuit voltage at room temperature and atmospheric pressure. Therefore, the function relationship of the open circuit voltage changing with air pressure is $V=V_{0}\cdot\left(1+\frac{N}{P}\right)$ (N is a constant) from abbreviated Equation (S3) at specified temperature. However, as different load resistance is often required in actual measurement, the relationship between output voltage under load resistance and air pressure need further correction.

The change of ambient air pressure has significantly effect on the total temperature change of pyroelectric element under radiation. According to the Equation (4), the output current *i*_p_ is mainly related to the change rate of sample temperature rather than the change of temperature, so it has a weak relationship with air pressure in the experimental conditions of this work. In view of the power expression of $P=i_{p}^{2}R$ (*R* is resistance), it can be inferred that there is a little correlation between the power and air pressure here.

**Supplementary References**

[1] C. R. Bowen, J. Taylor, E. LeBoulbar, et al. Pyroelectric materials and devices for energy harvesting applications. Energy Environ. Sci. 2014; 7: 3836-3856.
